# Investigation of a Capnometry Guided Respiratory Intervention in the Treatment of Posttraumatic Stress Disorder

**DOI:** 10.1007/s10484-021-09521-3

**Published:** 2021-09-01

**Authors:** Michael J. Ostacher, Eileen Fischer, Ellie R. Bowen, Jihun Lyu, Denishia J. Robbins, Trisha Suppes

**Affiliations:** 1 Veterans Affairs Palo Alto Health Care System, Palo Alto, CA USA; 2 Department of Psychiatry and Behavioral Sciences, Stanford University School of Medicine, Palo Alto, CA USA; 3 Department of Psychiatry, VA Palo Alto HCS, 3801 Miranda Avenue, Mail Code 151T, Palo Alto, CA 94304 USA

**Keywords:** Posttraumatic stress disorder (PTSD), Anxiety/anxiety disorders, Mindfulness/meditation, Biofeedback, CGRI

## Abstract

Evidence‐based treatments for posttraumatic stress disorder (PTSD), including psychotherapies and medications, have high dropout and nonresponse rates, suggesting that more acceptable and effective treatments for PTSD are needed. Capnometry Guided Respiratory Intervention (CGRI) is a digital therapeutic effective in panic disorder that measures and displays end-tidal carbon dioxide (EtCO_2_) and respiratory rate (RR) in real-time within a structured breathing protocol and may have benefit in PTSD by moderating breathing and EtCO_2_ levels. We conducted a single-arm study of a CGRI system, Freespira®, to treat symptoms of PTSD. Participants with PTSD (n = 55) were treated for four weeks with twice-daily, 17-min at-home CGRI sessions using a sensor and tablet with pre-loaded software. PTSD and associated symptoms were assessed at baseline, end-of treatment, 2-months and 6-months post-treatment. Primary efficacy outcome was 50% of participants having ≥ 6-point decrease in Clinician Administered PTSD Scale (CAPS-5) score at 2-month follow up. Tolerability, usability, safety, adherence and patient satisfaction were assessed. CGRI was well tolerated, with 88% [95% CI 74–96%] having ≥ 6-point decrease in CAPS-5 scores at 2-months post-treatment follow up. Mean CAPS-5 scores decreased from 49.5 [s.d. = 9.2] at baseline to 27.1 [s.d. = 17.8] at 2-months post-treatment follow up. Respiratory rate decreased and EtCO_2_ levels increased. Associated mental and physical health symptoms also improved. This CGRI intervention was safe, acceptable, and well-tolerated in improving symptoms in this study in PTSD. Further study against an appropriate comparator is warranted.

*Trial registration* Clinicaltrials.gov NCT#03039231.

## Introduction

Posttraumatic stress disorder (PTSD) is marked by symptoms of hyperarousal, difficulties with emotional regulation, negative affect, and autonomic dysfunction (American Psychiatric Association, 2013). The primary evidence-based treatments for PTSD are trauma-focused psychotherapies, non-trauma-focused psychotherapies, and medications, predominantly antidepressants. These therapies have had varying degrees of success in their effectiveness and tolerability, including high dropout and nonresponse rates (Bisson et al., 2013; Foa et al., 2018; Garcia et al., 2011; Ostacher & Cifu, 2019; Steenkamp et al., 2015; Watts et al., 2013). One mechanism by which PTSD symptoms might be mediated and which could be an area of therapeutic focus is in addressing CO_2_ (carbon dioxide) sensitivity, which has been shown to reduce symptoms of panic disorder (PD) (Kaplan et al., 2020; Meuret et al., 2008, 2009; Tolin et al., 2017). While provocation of panic attack symptoms during carbon dioxide challenge tests was initially reported for panic conditions, it has been subsequently reported in individuals with established PTSD (Kellner et al., 2018; Muhtz et al., 2011) and at risk for development of PTSD after trauma exposure (Telch et al., 2012). Since the characteristic of carbon dioxide hypersensitivity is shared by both conditions, extending the use of CGRI to a population with PTSD was logical and potentially valuable clinical tool given the lack of medication-free treatment options for PTSD.

The overlap between PD and PTSD is further elucidated in several publications. Joscelyne and colleagues identified substantial overlap in key somatic symptoms in samples of individuals with panic disorder and PTSD, with high rates of palpitations, shortness of breath, chest pain, and dizziness. Panic symptoms in PTSD sufferers were highly associated with intrusive traumatic memories (Joscelyne et al., 2012). An additional longitudinal study established a bi-directional relationship between PD and PTSD. Individuals with panic conditions have a heightened risk of subsequently developing PTSD, and individuals with PTSD have a heightened risk of developing panic attacks. (Berenz et al., 2019). These authors cite that approximately 70% of individuals with PTSD have co-morbid panic attacks. Prior literature has also proposed activation of trauma memory, catastrophic cognitions, culturally-specific associations, and interoceptive conditioning in response to fear sensations as possible panic attack triggers in PTSD sufferers. (Cougle et al., 2010).

This study aims to examine a novel intervention currently used to treat symptoms of PD. One marker of panic-related respiratory dysfunction is lowered end-tidal CO_2_ (EtCO_2_) levels (Gilbert, 2005; Roth, 2005). Capnometry Guided Respiratory Intervention (CGRI) is a digital therapeutic that gives users feedback of EtCO_2_ levels and respiration rate (RR), with the goal of increasing EtCO_2_ levels and decreasing RR. Foundational research has shown this to be effective in the treatment of PD by reducing symptoms that arise from breathing abnormalities (Meuret et al., , 2008, 2009). Subsequent studies have found one CGRI system (Freespira®) to be effective in reducing panic symptoms (Kaplan et al., 2020; Tolin et al., 2017). Given the symptomatic and epidemiological overlap between PTSD and PD noted above, it is plausible that the CGRI treatment, which has been shown to provide long-term improvement in PD, would lead to benefits in symptoms of PTSD. After recruitment for this study was completed, a study was published of a CGRI system that did not directly record participant use of the device found no differences compared to a waiting list control group in outcomes in Veterans with hyperarousal symptoms of PTSD (Jamison et al., 2019).

This study seeks to explore CGRI’s acceptability, tolerability, safety and effectiveness in reducing symptoms in patients with PTSD. We hypothesized in this open-label study that a 4-week at-home CGRI treatment, delivered for 17 min twice-daily in adult participants with moderate to severe symptoms of PTSD, would lead to a clinically significant reduction in PTSD symptoms 2 months post-treatment.

## Materials and Methods

### Participants

The study enrolled adults 18 years and older with a primary DSM-5 diagnosis of PTSD and who had a Clinician Administered PTSD Scale (CAPS-5) score of ≥ 30 (Weathers et al., 2013), a Clinical Global Impression-Severity (CGI-S) score of ≥ 4 (Guy, 1976), and who agreed (if on any psychotropic medication) to maintain their current stable dose from point of study entry until the 2-month post-treatment assessment. Subjects with additional DSM-5 disorders were able to be enrolled if PTSD was their primary psychiatric diagnosis.

Exclusions included: current evidenced-based therapy that focuses on PTSD (including cognitive processing therapy, EMDR, prolonged exposure therapy, virtual reality therapy, and cognitive behavioral therapy) during the treatment period and 2 month follow up and prior therapy must have been discontinued ≥ 1 month prior to enrollment; pregnancy; current enrollment in another device or drug study or enrollment in another drug or device study that was not at least 30 days past the final follow-up visit; suicidality, in the judgment of the interviewer; psychotic disorder, including schizophrenia and schizoaffective disorder; presence of uncontrolled bipolar disorder, including a manic episode in the past 6 months and not considered under control by the evaluator, or bipolar disorder is considered the primary diagnosis for the subject, in the interviewer’s opinion; alcohol or drug use disorder requiring acute medical treatment; epilepsy or recent seizures; and cardiovascular or pulmonary disease. Participants with a score of > 10 on the COPD assessment (CAT, Jones et al., 2009) or an EtCO_2_ of ≥ 48 mmHg at first treatment visit were excluded since they typically have impaired lung function due to pulmonary disease. Participants are required by the CGRI protocol to breathe to maintain to an EtCO_2_ of 40 mmHg, which is often not possible physiologically depending on lung disease severity. Participants were also excluded if unable to understand or comply with study procedures or if investigator determined that the subject was not eligible to participate in the study.

Participants were enrolled from February 2017 to February 2019, with recruitment from clinical settings in the VA Palo Alto Health Care System, along with posters and online advertising in the community. The trial was approved by the Stanford University Institutional Review Board and the VA Palo Alto Health Care System Research and Development Committee. Before enrollment, participants gave written informed consent to participate in the trial and were given a nine-question decisional capacity screen to confirm understanding of the consent.

### Procedures

After screening and written informed consent, potentially eligible subjects underwent baseline screening at our site, including a diagnostic assessment by a trained, Masters-level clinician, including review of medical and psychiatric history, current medications and substance use, CAPS-5 30-day version for confirmation of diagnosis and baseline score to confirm that PTSD was the primary diagnosis. The CAPS-5 is a 30-item clinician-administered scale that rates severity of PTSD symptoms drawn from DSM-5 criteria (Weathers et al., 2018). Secondary measures employed are as follows. The Patient Health Questionnaire 9-item depression scale (PHQ-9, Kroenke et al., 2001) is a self-report scale that asks individuals to rate the presence of DSM-IV symptom criteria ranging from ‘0’ (not at all) to ‘3’ (nearly every day). The Panic Disorder Severity Scale (PDSS, Shear et al., 2001, 1997), is a 7-item clinician-rated scale that indicates the severity and frequency of panic symptoms, fear of subsequent attacks, and avoidance behaviors. The Clinical Global Impression-Severity (CGI-S, Busner & Targum, 2007) is a single item clinician-rated measure of severity of psychopathology, using a 7-point Likert scale ranging from ‘normal’ to ‘among the most extremely ill patients’. The 36-Item Short Form Health Survey (SF-36, RAND Corporation, 2016; Ware, 1999) is a self-rated survey of health impact on daily function. The Concise Health Risk Tracking Self-Report (CHRT-SR, Ostacher et al., 2015; Trivedi et al., 2011) is a 12-item self-report inventory that assesses suicidal and related thoughts. The Chronic Obstructive Pulmonary Disease Assessment Test (CAT, Gupta et al., 2014) is an 8-item self-report scale that assesses the impact of COPD symptoms on function and quality of life. Study entry was reviewed by the study investigator (MJO).

Subjects were then trained on-site on the use of the CGRI device at the baseline visit and completed their first session at that time. Subsequent sessions were done at home. The CGRI device (Freespira®, Palo Alto Health Sciences, Inc. now Freespira, Inc.) trains individuals to regulate exhaled carbon dioxide levels (EtCO_2)_ and respiratory rate (RR) to meet specific goals for these parameters. Target RR was RR = 13 during week 1, RR = 11 during week 2, RR = 9 during week 3, and RR = 6 during week 4, and participants were instructed to adjust respiratory volume to attain or maintain a targeted EtCO_2_ of 40 mmHg. Normal resting RR is 12–15 breaths per minute (Folke, et al., 2003) and normal EtCO_2_ is > 35 mmHg (Oakes, 1996). CGRI equipment includes a proprietary handheld EtCO_2_ sensor, nasal cannula and tablet with pre-loaded software that guides the participant through two 17-min breathing sessions daily for 4 weeks. RR and EtCO_2_ were measured in real time for 2 min at rest at the start of each session to measure baseline readings and were continuously measured and displayed to the participant during the remaining 15 min of the session. Participants were instructed to breathe in sync with a rising and falling audio tone at the specified RR. De-identified data were securely uploaded to a server. Participants were evaluated in person or by phone weekly for the next four weeks. Outcome measures (30-day CAPS, PDSS, PHQ-9, SF-36, CHRT-SR, CGI-S) were evaluated at end of treatment, at 2-months post-treatment, and at 6-months post treatment. A questionnaire was provided to participants post-treatment and at 2- and 6-months to assess acceptability, usability and participant satisfaction. Adverse events were tracked throughout the study.

### Data Analytic Plan

Response was defined as a 6-point decrease in CAPS-5 score from baseline to 2-month follow up. The primary outcome was defined as ≥ 50% of study participants experiencing response between baseline and the 2-month follow-up. At the time of study initiation, during the change from the CAPS-IV (for DSM-IV) to the CAPS-5, a defined response score was not yet established for CAPS-5, thus we scaled the validated CAPS-IV response (Schnurr et al., 2007) of 10 by the ratio of maximum CAPS-5/CAPS-IV scores, 80/136 = 0.6, in order to identify a response score of 6 or more points. Later, the reliable change index (RCI) for CAPS-5 was defined as 13 points (Sloan et al., 2018) and was applied to the data retrospectively to provide additional validity of the CAPS-5 results in this study. This was a pilot trial, so we decided a priori that if more than half of the participants met the response criteria, then this at-home, non-invasive treatment might be worth pursuing for further study in this difficult to treat population. The 50% response was a clinical decision based on prior studies with CGRI and the limited effectiveness of current PTSD treatments in the context of the debilitating effects of PTSD. Proportions of participants with the desired outcome and associated 95% lower bounds were estimated. The proportions were also compared to 50% using a z-statistic test.

Remission was predefined as response plus no longer meeting DSM-5 criteria for PTSD and having a CAPS-5 score < 25. For the continuous outcomes (change from pre-treatment on CAPS-5, PDSS, CHRT-SR, SF-36, PHQ-9, CGI-S, Baseline EtCO_2_ and Baseline RR), the mean score change was estimated, and Cohen’s d effect size was calculated using the sample standard deviation of the mean difference. Descriptive statistics for RR and EtCO_2_ were analyzed for changes over the course of the study. Given the exploratory nature of the study, adjustment was not made for multiple comparisons.

The protocol’s target completer sample-size of 44 was identified to allow the lower 95% confidence limit of the proportion achieving a successful response to be 35%, from an expected response rate of 50%. We expected the response rate for the primary outcome to equal or exceed 50% based on prior studies in PD. Using the normal approximation to the binomial distribution, a sample of 44 participants was required to be 95% confident that the difference between the true and observed rates differed by no more than ± 15%. Allowing for a dropout rate of 20%, a total of 55 participants were planned.

Baseline characteristics included all participants who completed the baseline session. Analyses of outcomes were conducted using a modified intention-to-treat approach. For participants with at least one post-baseline data point (n = 48), CAPS-5, PDSS, CHRT-SR, PHQ-9, SF-36 and CGI-S scores at post-treatment and at 2- and 6-month follow-up were estimated based on previous values using an iterative Markov chain Monte Carlo (MCMC) method. The imputation for missing values was repeated five times. The corresponding statistical method was applied to each of the five imputed data sets and results were averaged across imputed data sets appropriately accounting for the between and within imputed data set variances. All analyses were performed using SPSS (IBM) v27.

## Results

55 participants completed the baseline assessment, of which 36 (65%) were male and 19 female (35%), with a mean age of 51 years (range 19–77) and with 39 (71%) military veterans and 16 (29%) civilians. Source of primary trauma was combat-related in 16 (29%) of participants and sexual assault in 18 (33%) of participants with additional index trauma reported as accidents (3), family member suicide (2), death in family (3), physical abuse (4) and other (2). Past panic attacks were reported by 40 (73%) participants, with lifetime major depressive disorder in 27 (50%) and lifetime bipolar disorder in 10 (18%). Lifetime psychiatric hospitalization was reported by 21 (39%) of participants.

**Fig. 1:**
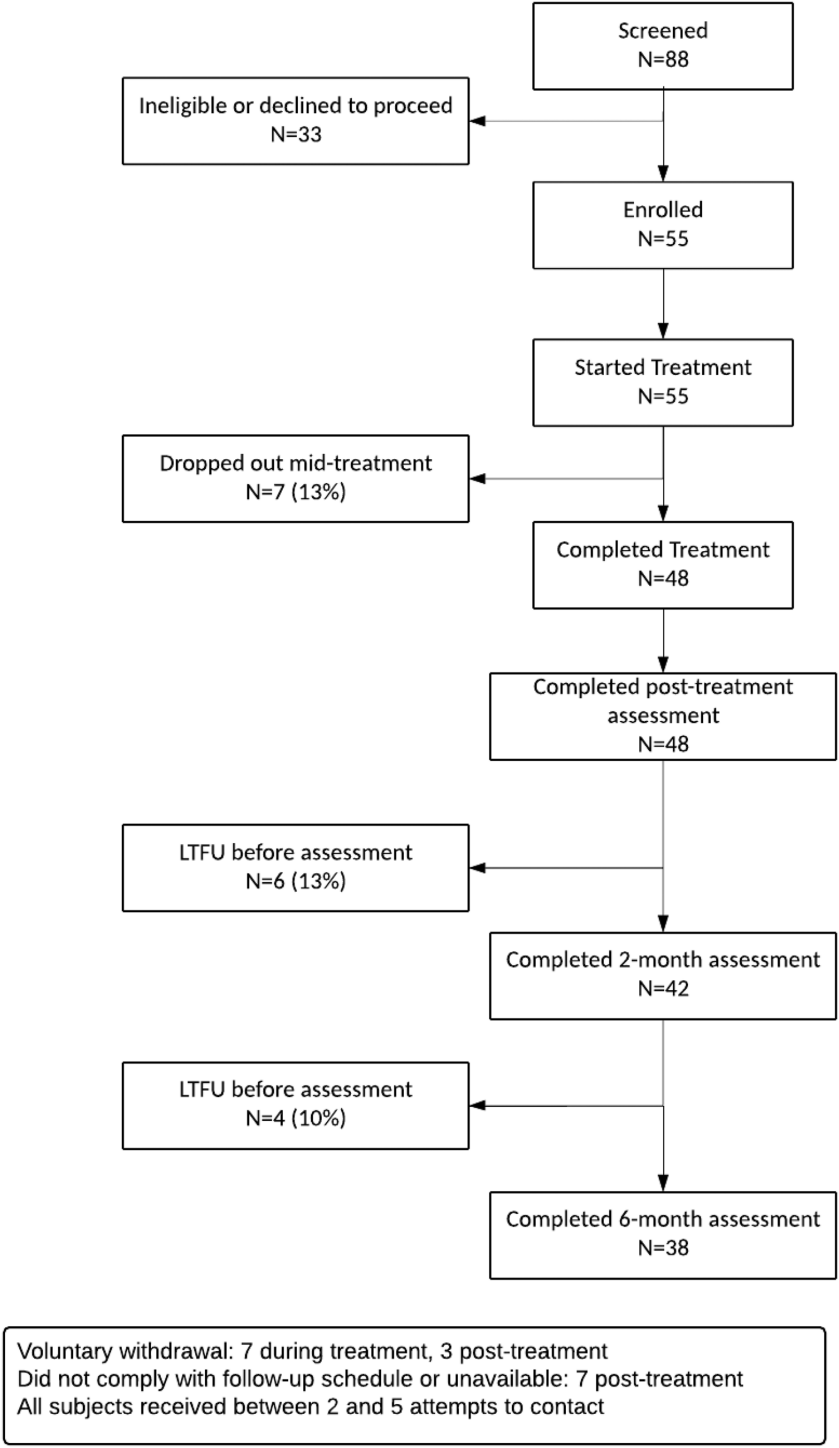
Participant disposition

Baseline demographics are seen in Table 1. Disposition of the participants is shown in Fig. 1.

**Table 1:** Sample description

Baseline study demographics	
N	55
Age: M (SD)	51 (14)
Veteran N (%)	39 (71%)
Sexual assault related PTSD N (%)	18 (33%)
Combat related PTSD N (%)	16 (29%)
Male N (%)	36 (65%)
Ethnicity:	
African American N (%)	15 (28%)
Caucasian N (%)	23 (43%)
Native Hawaiian/Pacific Islander N (%)	2 (4%)
American Indian/Alaska Native N (%)	6 (11%)
Asian N (%)	2 (4%)
Mixed N (%)	3 (6%)
Decline-to-state N (%)	4 (7%)
Comorbid medical conditions	
Parkinson’s N (%)	0 (0%)
Alzheimer’s N (%)	0 (0%)
Stroke N (%)	3 (5%)
Other N (%)	7 (13%)
Seizures	
Childhood fever N (%)	2 (4%)
Adult-onset N (%)	1 (2%)
Head trauma or loss of consciousness N (%)	21 (38%
Cardiovascular	
Heart disease N (%)	1 (2%)
Heart failure N (%)	1 (2%)
Kidney failure N (%)	1 (2%)
Respiratory	
Asthma N (%)	4 (7%)
COPD N (%)	0 (0%)
Emphysema N (%)	1 (2%)
Chronic bronchitis N (%)	2 (4%)
Endocrine	
Hypo or hyperthyroid N (%)	6 (11%)
Diabetes N (%)	9 (16%)
Care	
Regularly see a GP or Specialist N (%)	48 (87%)
Lifetime inpatient psychiatric hospitalization	21 (39%)
Current suicidal ideation N (%)	3 (6%)
Previous mental health diagnosis:	
Bipolar Disorder N (%)	10 (18%)
Schizophrenia N (%)	0 (0%)
Autism N (%)	0 (0%)
Asperger’s N (%)	0 (0%)
Major depression N (%)	27 (50%)
PTSD N (%)	47 (85%)
Hospitalization	
Hospitalization in last 12-months N (%)	12 (22%)
ER visit in last 12-months N (%)	20 (36%)
Habits	
Drink alcohol N (%)	28 (51%)
Smoke N (%)	14 (25%)
Recreational drugs N (%)	10 (19%)
Panic attacks along with PTSD N (%)	40 (73%)
Years experienced panic attacks w/PTSD M (SD)	19 (14)
Currently seeking treatment for PTSD N (%)	45 (82%)
Previously sought treatment for PTSD N (%)	44 (80%)
Currently seeking treatment for panic attacks N (%)	27 (49%)
Previously sought treatment for panic attacks N (%)	31 (56%)

Evaluation of the primary outcome measure (≥ 6 point drop in CAPS-5 score from baseline to 2-month post-intervention follow up, prespecified at baseline as a clinically significant outcome, and positive for the trial if ≥ 50% of subjects reached remission) showed 88% of subjects (95% CI 74–96) reaching criteria for response, with 48% of subjects reaching criteria for remission. CAPS-5 reductions far exceeded the 6-point reduction endpoint, with mean CAPS-5 scores decreasing from 49.5 (s.d. = 9.2) at baseline to 27.1 (s.d. = 17.8) at 2-months, with an effect size d′ = 1.3.

This represents a 48% reduction of the CAPS-5 score. Similar changes were seen immediately post-treatment and persisted through 6-month post-treatment follow-up. We also calculated the reliable change index (RCI) as further confirmation of study results. Using the baseline (49.5) and 2-months (27.1) CAPS-5 scores and the SE of the difference between baseline and 2-month follow-up (2.5) the CAPS-5 reliable change index is 9 points. Using this study’s RCI of 9-points and Sloan’s RCI of 13-points definition for response, 83% and 76% of subjects met criteria at 2-months and 87% and 82% meet response criteria at 6-months.

Decreases were seen in the PDSS, PHQ-9, CHRT-SR, and CGI-S at 2-month follow up, with maintenance of improvement at 6 months, and the SF-36 sub-scale ‘role functioning /emotional’ showed a large effect size, with improvement continuing to 6 months. Decreases in CAPS-5 and other outcomes scores (including effect sizes) are shown in Table 2.

**Table 2:** Outcomes on primary and secondary measures

	Pre	Post			2-Month follow-up			6-Month follow-up		
	M (SD)	M (SD)	Est. mean change (SE)	d’	M (SD)	Est. mean change (SE)	d′	M (SD)	Est. mean change (SE)	d′
CAPS-5	49.5 (9.2)	31.8 (14.1)	17.7 (1.9)	1.4	27.1 (17.8)	22.4 (2.5)	1.3	26.2 (18.4)	23.4 (2.4)	1.4
PDSS	9.9 (5.4)	7.5 (6.4)	2.4 (0.9)	0.4	6.0 (6.4)	3.9 (1.0)	0.6	5.2 (7.3)	4.7 (1.1)	0.7
CHRT-SR	15.4 (8.8)	11.3 (9.7)	4.2 (1.2)	0.5	11.0 (10.1)	4.5 (1.3)	0.5	10.4 (9.3)	5.0 (1.4)	0.5
PHQ-9	14.7 (4.9)	10.8 (7.1)	4.0 (0.9)	0.6	11.8 (6.9)	2.9 (1.0)	0.4	10.2 (6.5)	4.5 (1.0)	0.6
CGI-S	4.8 (0.6)	3.2 (1.3)	1.6 (0.2)	1.2	3.0 (1.6)	1.9 (0.2)	1.2	3.0 (1.8)	1.8 (0.3)	1.0
SF-36^3^	13.2 (27.3)	37.5 (40.5)	24.3 (5.7)	0.6	37.1 (43.0)	23.9 (6.6)	0.5	43.9 (39.6)	30.7 (6.1)	0.7
ETCO_2_	36.2 (4.3)	37.9 (4.3)	1.7 (0.7)	0.3						
RR	14.9 (3.9)	12.8 (6.3)	2.1 (0.7)	0.4						

Mean EtCO_2_ increased modestly from baseline to end of treatment, with a mean of 36.2 (s.d. = 4.3) at baseline, increasing to 37.9 (s.d. = 4.3) at end-of-treatment, while RR showed moderate signs of decrease from pre-treatment to post-treatment, with a mean of 14.9 (s.d. = 3.9) at baseline, decreasing to 12.8 (s.d. = 6.3) at end-of-treatment.

To further investigate the effect of treatment on EtCO_2_, subjects were analyzed separately based on baseline hypocapnia (EtC0_2_ < 36 mmHg) or normocapnia. Within the hypocapnic group (n = 21), EtCO_2_ increased from 32.4 (s.d. = 2.4) to 36.3 (s.d. = 5.4) whereas the normocapnic group (n = 27) exhibited a much smaller change from 39.4 (s.d. = 2.5) to 39.1 (s.d. = 4.9). For the hypocapnic group, the improvement in CAPS-5 score was also greater (d′ = 1.9 at post-treatment) vs. a smaller effect (d′ = 0.7 post-treatment) for normocapnic subjects. See Table 3.

**Table 3:** Outcomes split by Hypo/Normocapnic at baseline

	Pre	Post			2-Month follow-up			6-Month follow-up		
	M (SD)	M (SD)	Est. mean change (SE)	d′	M (SD)	Est. mean change (SE)	d′	M (SD)	Est. mean change (SE)	d′
Hypocapnic (Pre ETCO2 < 36 mmHg, n = 21)										
CAPS-5	47.8 (5.8)	25.3 (10.5)	22.5 (2.2)	1.9	21.9 (19.1)	26.0 (4.3)	1.3	17.4 (12.1)	30.4 (2.8)	2.3
ETCO_2_	32.4 (2.4)	36.3 (5.4)	3.9 (1.3)	0.7						
RR	15.0 (4.2)	13.6 (7.3)	1.4 (1.1)	0.2						
Normocapnic (Pre ETCO ≥ 36 mmHg, n = 27)										
CAPS-5	51.1 (11.3)	37.1 (15.3)	14.1 (3.0)	0.7	31.2 (16.2)	19.9 (3.2)	1.0	32.2 (19.2)	18.9 (3.4)	0.8
ETCO_2_	39.4 (2.5)	39.1 (4.9)	0.3 (0.9)	0.1						
RR	14.8 (3.8)	13.3 (6.5)	1.5 (1.1)	0.2						

*CAPS-5* Clinician Administered PTSD Scale, *PDSS* Panic Disorder and Severity Scale, *CHRT-SR* Concise Health Risk Tracking Self-Report, *PHQ-9* Patient health Questionnaire 9-item depression scale, *CGI-S* Clinician Global Impression—Severity score, *SF-36*^*3*^ 36-item Short Form Health Survey question 3 (Role functioning/emotional), *ETCO*_*2*_ End-tidal carbon dioxide (mmHg), *RR* Respiration Rate (breaths per minute, bpm)

For all subjects who completed > 14 sessions, adherence to the 4-week protocol was calculated by determining the proportion of CGRI sessions completed over the course of the study (target = 56), as evidenced by automatic uploads to the cloud-based server. Adherence was calculated as the number of completed sessions divided by 56 (capped at 100%), consistent with previous CGRI studies (Kaplan et al., 2020; Tolin et al., 2017). The overall mean adherence using this calculation was 77% (43/56).

Participants completed a questionnaire post-treatment to assess acceptability, usability and participant satisfaction. Of the 47 completed surveys, 83% said they ‘would’ or ‘would definitely’ recommend this treatment to a friend or family member, and 76% said they were ‘satisfied’ or ‘very satisfied’ with their treatment. Seventy percent said it was ‘easy’ or ‘very easy’ to learn and use the device. When asked to rank the benefits of Freespira in order of importance, 73% chose as #1 or #2, ‘I could use it at home’. When asked how easy it was to make time to do the sessions twice per day for 28 days, 39% said it was ‘easy’ or ‘very easy’, while 36% said it was ‘difficult’ or ‘very difficult’ to find time. The ‘recommend to a friend’ and satisfaction responses remained stable or slightly increased at 2 and 6 months.

There were 14 adverse events reported; 10 were determined “not related” to the treatment/device and 4 were “possibly related”. Of the four, two occurred during the course of treatment: chest pain on the last day of use, which resolved without action being taken, and shortness of breath at night, which also resolved without treatment and had been experienced before study enrollment. Participants in this study did not report dizziness or light-headedness during the early sessions as had been reported in a trial of the device for PD.

## Discussion

In this open-label, single-arm study of four weeks of a twice daily at-home CGRI, participants with PTSD showed a marked decrease in CAPS-5 scores at the prespecified primary endpoint of two-month follow-up after treatment completion. Overall, there was a clinically significant reduction in PTSD scores over the course of the study and decreases in CAPS-5 scores persisted (and continued to decline) at six-month follow-up, with 50% meeting the criteria for remission at 6 months. The device was safe, participants reported that it was easy to learn and use, and while 36% of the participants had some difficulty finding time to do the twice-daily sessions, adherence to the assigned protocol over four weeks was quite good, thus consistent with the durability of outcomes.

After four weeks of treatment there was an increase in EtCO_2_ levels and a decrease in RR compared to baseline, which suggests physiological and as well as behavioral changes associated with the use of the device. Prior research in panic disorder demonstrates physiological changes associated with CGRI, with perceived control playing an additional role in symptom reduction (Meuret et al., 2010).

The results of this study show promise of this digital therapeutic, but the mechanism by which CGRI improves PTSD symptoms merits additional investigation. The subjects in this study who were hypocapnic increased their EtCO_2_ and the magnitude of their symptom improvement was greater than the normocapnic group. Normalization of baseline EtCO_2_ is present in some but not all treatment responders, as 56% were within the normal range at baseline and remained so at follow up, as seen in other studies of CGRI in panic disorder (Kaplan et al., 2020; Meuret et al., 2008; Tolin et al., 2017). What is not fully known at this point is whether the learned breathing style taught during the CGRI protocol enables users to suppress dysregulated breathing when exposed to ‘triggering’ situations and engage instead in the trained breathing pattern as they become aware of physiological and/or psychological distress. CGRI’s repetitive training of paced/normocapnic breathing likely develops self-management skills that can be deployed at awareness of symptom increase. As with many therapies, the mechanism of action is unlikely to be unitary. In the case of CGRI, respiratory stability, expectancy, and development of an overlearned skill (paced breathing with normal tidal volume) accessible during times of stress all likely contribute to the treatment response.

A recent publication of a trial of Capnometry Assisted Respiratory Training (CART) targeting PTSD hyperarousal in Veterans failed to find a benefit from CART compared to a waiting list control group using CAPS-IV hyperarousal scores as an outcome (Jamison et al., 2019). The magnitude of the benefit in the earlier trial was smaller for both the intervention and the waitlist control compared to the changes found in our study. There are a number of differences in these trials that may explain the differences in outcomes. The Jamison study, which was conducted with different instrumentation, exclusively used a RR target of 9 over four weeks, compared to 13, 11, 9 and 6 RR targets decreasing weekly over the 4-week intervention using the device in this study, which may have had an impact on outcomes. The Jamison trial also had higher dropout rates, and actual adherence to the twice-daily 17-min breathing sessions were not measured or known in real-time but rather required that the participants document their performance and measures in a written log. In our trial of an integrated and internet-connected system, the data from each session were recorded in real-time and uploaded to a server, and participants received weekly coaching about their adherence and success in meeting RR and EtCO_2_ targets. Thus, it is possible that the protocol used in this study was more engaging for the user and may have led to better adherence and more actual use of the device as intended. The strengths of our study are that it included subjects with well-characterized PTSD, was performed in an academic medical center with the accompanying oversight, had broad inclusion and narrow exclusion criteria, did not require or limit the concurrent use of medications, and included both military veterans and civilians with this disorder. Completion rates, acceptability, adherence, usability, safety and effectiveness of the treatment were high.

The primary limitation of the present study is the absence of a randomly assigned comparison group. As this was a first-in-illness study of this specific device, the study was powered to determine whether clinically meaningful improvement of symptoms would be found. It is not known to what extent study participation itself or other performance biases might have led to such marked improvements in CAPS-5 outcomes. Also, this study enrolled more men than women, with a substantial proportion of participants having combat trauma, so the generalizability of the results to either a purely military veteran or purely civilian population is not known. In addition, the psychiatric comorbidity (in terms of comorbid diagnosis and past hospitalization rates) was high, so the generalizability to a less ill population is also not known but suggests that the device will have benefit even in patients with significant mental health comorbidities.

## Conclusion

In this trial of a CGRI device (Freespira®) in a mixed population of military veterans and civilians with PTSD, four weeks of 17-min twice-daily at-home breathing training sessions led to a marked decrease in PTSD scores for the majority of participants as measured by the CAPS-5 at 2-month post-intervention follow up. Remission rates were significant at 2-months and 6-months post-treatment. Associated measures of mental and physical health also improved and persisted at 6-month follow-up.

There is a need for safe, tolerable, acceptable, and effective treatments for the symptoms of PTSD that can be easily and widely disseminated for use in a patient’s home, including to rural residents who have difficulty accessing in-person services. This proprietary CGRI system is a four-week intervention that was acceptable to participants, is without the adverse effects of pharmacological interventions, does not require the intervention of trained staff, and may be more accessible and tolerable than trauma-focused or other face-to-face psychotherapies. The combination of clinical improvement to six months and high adherence and participant satisfaction rates suggests that this non-invasive, digital therapeutic is a promising intervention for PTSD. Further study of this CGRI system for PTSD against an appropriate comparator treatment is warranted.
